# Supplementary material for: Thirty-day readmission after medical-surgical hospitalization for people who experience imprisonment in Ontario, Canada: A retrospective cohort study
Source: PLoS One. 2020 Jan 10;15(1):e0227588. doi: 10.1371/journal.pone.0227588 (PMC6953830; doi:10.1371/journal.pone.0227588)
Supplement: S1 Table — (DOCX) [file pone.0227588.s001.docx]

**S1 Table. Most common case mix groups for the index hospitalization among prison group,* N=1,548**

| Case Mix Group | People in prison,  N=262 | People recently released from prison, N=1,286 |
| --- | --- | --- |
| Poisoning/Toxic Effect of Drug | 9 | 109 |
| Psychoactive Substance Use, Withdrawal State | 13 | 33 |
| Internal Fixation of Facial Bone | 14 | 25 |
| Seizure Disorder, except Status Epilepticus | 8 | 29 |
| Open Wound/Other/Unspecified Minor Injury | † | 33 |
| Diabetes | 6 | 27 |
| Cellulitis | † | 27 |
| Psychoactive Substance Use, Acute Intoxication | † | 29 |
| Gastrointestinal Hemorrhage | † | 25 |
| Angina (except Unstable)/Chest Pain without Cardiac Catheter | 7 | 19 |
| Disorder of Pancreas except Malignancy | † | 25 |
| Convalescence | † | 24 |
| Reduction/Fixation/Repair of Ankle/Foot | † | 21 |
| Replacement/Fixation/Repair of Tibia/Fibula/Knee | † | 20 |
| Symptom/Sign of Digestive System | 6 | 16 |
| Fracture of Skull/Facial Bone | † | 16 |
| Single Injury to Internal Organ | † | 18 |
| Major Thoraco-abdominal/Vascular Intervention with Trauma/Complication of Treatment | † | 16 |
| Reduction/Fixation/Repair Upper Body/Limb except Fixation/Repair of Shoulder | † | 15 |
| Viral/Unspecified Pneumonia | † | 14 |
| Chronic Obstructive Pulmonary Disease | † | 15 |
| Esophagitis/Gastritis/Miscellaneous Digestive Disease | † | 13 |
| Abscess | † | 12 |
| Cardiac Valve Disease | † | 11 |
| Cirrhosis/Alcoholic Hepatitis | † | 12 |
| Single Intracranial Injury | † | 10 |
| Other | 147 | 672 |

*People released from provincial prison in Ontario in 2010 who were admitted to hospital between 2005 and 2015 while in provincial prison or within 6 months of release from provincial prison. †For cells with n ≤5, we suppressed the number as per ICES policy.
