# Supplementary material for: Thirty-day readmission after medical-surgical hospitalization for people who experience imprisonment in Ontario, Canada: A retrospective cohort study
Source: PLoS One. 2020 Jan 10;15(1):e0227588. doi: 10.1371/journal.pone.0227588 (PMC6953830; doi:10.1371/journal.pone.0227588)
Supplement: Attachment — Submitted filename: Response to reviewers October 22 2019.docx [file pone.0227588.s003.docx]

Response to reviewers

**Journal Requirements:**

*When submitting your revision, we need you to address these additional requirements.*

*1. Please ensure that your manuscript meets PLOS ONE's style requirements, including those for file naming. The PLOS ONE style templates can be found at*

*http://www.journals.plos.org/plosone/s/file?id=wjVg/PLOSOne_formatting_sample_main_body.pdf and http://www.journals.plos.org/plosone/s/file?id=ba62/PLOSOne_formatting_sample_title_authors_affiliations.pdf*

We have reviewed these documents and revised the manuscript and supplementary files to meet PLOS ONE’s style requirements.

*2. In ethics statement in the manuscript and in the online submission form, please provide additional information about the patient records used in your retrospective study. Specifically, please ensure that you have discussed whether all data were fully anonymized before you accessed them and/or whether the IRB or ethics committee waived the requirement for informed consent. If patients provided informed written consent to have data from their medical records used in research, please include this information.*

We have added a section regarding the ethics review at the end of the Methods section. We have specified in that section that we used nominal data only for data linkage purposes and that the requirement for informed consent was waived.

*3. Please correct your reference to "p=0.000" to "p<0.001" or as similarly appropriate, as p values cannot equal zero.*

We have revised this text in Table 1.

*4. We noticed you have some minor occurrence of overlapping text with the following previous publication(s), which needs to be addressed:*

*- Kouyoumdjian, Fiona G., et al. "The health care utilization of people in prison and after prison release: A population-based cohort study in Ontario, Canada." PloS one 13.8 (2018): e0201592.
Khanna, Sumeet, et al. "Health care utilization by people with HIV on release from provincial prison in Ontario, Canada in 2010: a retrospective cohort study." AIDS care 31.7 (2019): 785-792.*

*The text that needs to be addressed involves the first paragraph of the Introduction.*

*In your revision ensure you cite all your sources (including your own works), and quote or rephrase any duplicated text outside the methods section. Further consideration is dependent on these concerns being addressed.*

We have made revisions to the first paragraph of the Introduction based on comments from the reviewers, as described subsequently in this response. We have reviewed all citations to ensure that we have appropriately referenced all work, include previous work by members of our research group.

We have reviewed the first paragraph of the Introduction of this manuscript and compared that content with the content in the two articles noted. We would be happy to further revise the content or to add additional references to any content that is unacceptably similar to content in other published articles by our research group.

*5. We note that you have indicated that data from this study are available upon request. PLOS only allows data to be available upon request if there are legal or ethical restrictions on sharing data publicly. For information on unacceptable data access restrictions, please see http://journals.plos.org/plosone/s/data-availability#loc-unacceptable-data-access-restrictions.*

*In your revised cover letter, please address the following prompts:*

*a) If there are ethical or legal restrictions on sharing a de-identified data set, please explain them in detail (e.g., data contain potentially identifying or sensitive patient information) and who has imposed them (e.g., an ethics committee). Please also provide contact information for a data access committee, ethics committee, or other institutional body to which data requests may be sent.*

*b) If there are no restrictions, please upload the minimal anonymized data set necessary to replicate your study findings as either Supporting Information files or to a stable, public repository and provide us with the relevant URLs, DOIs, or accession numbers. Please see http://www.bmj.com/content/340/bmj.c181.long for guidelines on how to de-identify and prepare clinical data for publication. For a list of acceptable repositories, please see http://journals.plos.org/plosone/s/data-availability#loc-recommended-repositories.*

*We will update your Data Availability statement on your behalf to reflect the information you provide.*

We are unfortunately not able to share the de-identified data set. Our Research Agreement with the Ministry of Community Safety and Correctional Services (MCSCS) specifies that “The Researchers…shall not reproduce or otherwise make copies of Data including records, or any parts thereof…without first obtaining the written permission of the Ministry.”

We suggest the following statement (similar to the Data Availability statement provided for PLOS ONE article “The health care utilization of people in prison and after prison release…,” https://journals.plos.org/plosone/article?id=10.1371/journal.pone.0201592):

We are not able to share data because of restrictions specified in our Research Agreement with the Ministry of Community Safety and Correctional Services and in the data sharing agreements of ICES. People who would like access to data from the Ministry should direct requests to Michael Kirk at [Michael.kirk@ontario.ca](mailto:Michael.kirk@ontario.ca). Access to data at ICES can be granted to those who meet pre-specified criteria for confidential access, available at [www.ices.on.ca/](http://www.ices.on.ca/). Requests to access ICES data for research purposes may be submitted to ICES’ Data and Analytic Services, with information available at <http://www.ices.on.ca/DAS> and contact: [das@ices.on.ca](mailto:das@ices.on.ca).

6. *Thank you for stating the following in the Acknowledgments Section of your manuscript:*

*"This study was supported by ICES, which is funded by an annual grant from the Ontario Ministry of Health and Long-Term Care. The opinions, results and conclusions reported in this paper are those of the authors and are independent from the funding sources. We acknowledge the Ontario Ministry of Community Safety and Correctional Services, which provided data for the study. No endorsement by ICES, the Ontario Ministry of Health and Long-Term Care, or the Ministry of Community Safety and Correctional Services is intended or should be inferred. Parts of this material are based on data and/or information compiled and provided by CIHI. However, the analyses, conclusions, opinions and statements expressed in the material are those of the authors, and not necessarily those of CIHI.*

*We note that you have provided funding information that is not currently declared in your Funding Statement. However, funding information should not appear in the Acknowledgments section or other areas of your manuscript. We will only publish funding information present in the Funding Statement section of the online submission form.*

*Please remove any funding-related text from the manuscript and let us know how you would like to update your Funding Statement. Currently, your Funding Statement reads as follows:*

*"FK received funding from Physicians' Services Incorporated Foundation (15-22)."*

To clarify, we did not receive any funding from ICES; the text specified in the Acknowledgments section is standard text that studies conducted at ICES are required to specify, but the “support” reflects in kind support rather than funding. We also did not receive any funding from the other institutions that we acknowledged.

The funding source specified in the Funding Statement is the only funding source for this study, and therefore we do not think we need to update the Funding Statement.

**Reviewers' comments:**

**Comments to the Author**

1. Is the manuscript technically sound, and do the data support the conclusions?

The manuscript must describe a technically sound piece of scientific research with data that supports the conclusions. Experiments must have been conducted rigorously, with appropriate controls, replication, and sample sizes. The conclusions must be drawn appropriately based on the data presented.

Reviewer #1: Partly

Reviewer #2: Yes

2. Has the statistical analysis been performed appropriately and rigorously?

Reviewer #1: Yes

Reviewer #2: Yes

3. Have the authors made all data underlying the findings in their manuscript fully available?

The [PLOS Data policy](https://macmail.mcmaster.ca/owa/redir.aspx?C=rg0VRKa8r0EkMLTx9sXm9uZawJjgVZvqbG0ytAcWvpt1jHv8NzvXCA..&URL=http%3a%2f%2fwww.plosone.org%2fstatic%2fpolicies.action%23sharing) requires authors to make all data underlying the findings described in their manuscript fully available without restriction, with rare exception (please refer to the Data Availability Statement in the manuscript PDF file). The data should be provided as part of the manuscript or its supporting information, or deposited to a public repository. For example, in addition to summary statistics, the data points behind means, medians and variance measures should be available. If there are restrictions on publicly sharing data—e.g. participant privacy or use of data from a third party—those must be specified.

Reviewer #1: No

Reviewer #2: No

4. Is the manuscript presented in an intelligible fashion and written in standard English?

PLOS ONE does not copyedit accepted manuscripts, so the language in submitted articles must be clear, correct, and unambiguous. Any typographical or grammatical errors should be corrected at revision, so please note any specific errors here.

Reviewer #1: Yes

Reviewer #2: Yes

5. Review Comments to the Author

Please use the space provided to explain your answers to the questions above. You may also include additional comments for the author, including concerns about dual publication, research ethics, or publication ethics. (Please upload your review as an attachment if it exceeds 20,000 characters)

*Reviewer #1: This manuscript investigates the readmission and return to acute care after hospitalization among persons who experienced incarcerations and persons from the general population. This is an important research question, as the released prisoners constitute an underserved population and because their health care use is not well understood. The manuscript relies on a large sample size with a sound methodology. However, there are some limitations that should be addressed.

Whole paper
1. The literature more commonly uses “emergency department visits” rather than “return to acute care”.*

We defined return to acute care as either emergency department visits or hospital readmission. However, after reflecting on feedback from the reviewers, we have decided to focus the manuscript only on hospital readmission as the outcome of interest. So we no longer use the term return to acute care in the manuscript.

*Abstract
2. I found the abstract very confusing at first read. I am afraid it is not readable as a stand-alone piece. It should be written more clearly. The results section is very long and the conclusions are almost absent. I did not understand the Methods at first read.*
We have substantially revised the Abstract to improve clarity, including shortening the Results section. We hope that the revised Abstract is now more clear.

*Introduction
3. The introduction is very short. There are some studies addressing health care (and use of emergency department) among (released) prisoners. This literature should be presented as well. As it is not possible to understand the reasons of readmission or return to acute care, I suggest to avoid mentioning it at the first place in the Introduction.*

We have referenced several studies that examined health care use for people after release from prison.

We agree that we are not able to assess the reasons for readmission rates in this paper, which we have discussed in the limitations section of the Discussion. However, we think it is nonetheless important to discuss the potential reasons for readmission, since this represents the justification for assessing the readmission rates for this population. For example, high readmission rates might indicate poor quality of care in hospital, and that provides one justification for defining readmission rates in this study. We hope to conduct research in the future to explore reasons for decreased readmission rates in people after prison release.

*Methods
4. The most important shortcoming is the presence of the incarcerated prisoners and the potential lack of power. The non-significant findings are not interpretable and there are important issues (prisoners possibly released). I suggest to remove this group from the sample and to focus on released prisoners and general population.*
We agree that the analysis of people in prison likely lacks sufficient power, and we have discussed this in the limitations section of the Discussion. However, we think it is valuable to present the findings nonetheless, especially given that this is the first study to explore rates of readmission in this population. We have specified in the limitations section that the findings of the analyses of people in prison should be considered exploratory.

*5. What about released prisoners who are reincarcerated within 6 months?*

For the index event, we included any medical-surgical hospitalization that occurred while a person was in prison or within 6 months of any prison release. When we looked at the outcome of medical-surgical readmission over 30 days, we included the full 30-day period, whether people were released from prison (if in prison at the time of index hospital discharge) or re-incarcerated (if recently released at the time of index hospital discharge) during the 30 days. We have expanded on the discussion of this issue in the limitations paragraph of the Discussion.

*6. Using a random hospitalization appears as a limitation of the study design. The rationale for this choice instead of using the first hospitalization should be given.*

We decided to use a random hospitalization in order to calculate the average risk of the outcome of readmission for any single hospitalization across people over the follow up period. The risk of readmission may have been higher or lower if we had instead selected either the first or last hospitalization, and if there are temporal or age-related trends in reasons for hospitalization, including the first or last hospitalization might over-represent specific reasons for hospitalization rather than reflecting all reasons for hospitalization over this period.

We have discussed the random selection of a hospitalization in the third paragraph of the Exposure section of the Methods, as well as in the limitations paragraph of the Discussion.

*7. Please add detailed information on variables assessed in the study.*

We have added additional information on variables in the Exposure, Covariates, and Outcome sections of the Methods. We have provided references when possible for all variables.

*8. Please also give a rationale for using a 30-day readmission or return to acute care cut-off.*

We selected a 30-day follow up period for the outcome of readmission because this period is commonly used as an indicator of quality of patient hospital care, e.g. by the Centers for Medicare & Medicaid Services, as discussed in several references, including reference #1 by Axon et al. and reference #2 by Joynt *et al.* We appreciate that there has been debate about the value of this outcome, and in particular it has been noted that readmissions within 3 or 7 days after hospital discharge may more accurately reflect quality of care in hospital compared with readmissions within 30 days of hospital discharge, as discussed in reference #2 by Joynt *et al.*

*Results
9. The 7-day outcome is mentioned for the first time p. 9 in the Results section. It should be included in the study’s objective with its rationale and in the Methods section.*

Thank you for pointing this out. We have decided to cut content regarding the 7-day period, since our focus is on 30-day readmission rates and our regression analyses are focused on the 30-day period, and in consideration of sample size issues.

*10. An important missed variable is the reason for hospitalization. It should be controlled for to achieve a better understanding of the results.*

We agree that it would be interesting to explore reasons for readmission to hospital. However, we think it would be outside of the scope of this study to conduct those analyses, as discussed in the limitations section of the Discussion. In this study, our goal was to compare rates of 30-day hospital readmission for people who experience imprisonment and matched people in the general population. In subsequent work, we hope to further explore reasons why people were and were not readmitted to hospital.

*Discussion
11. Released prisoners are more likely to visit emergency departments in comparison with the general population. This may be because they have no GP, no information on their own health insurance, or no money to pay extra fees. It does not mean that they have worse outcomes after hospitalization. This should be discussed in the paper. Information on health care fees in the Canadian health care system should also be included.*
We agree that ED visits in people recently released from prison may be caused by several reasons. As noted above, we have removed return to acute care as an outcome from the paper.

We have added content in the fifth paragraph of the Discussion regarding the increased rate of ED visits as a potential reason for the decreased readmission rate in people recently released from prison.

We have added content in the Setting section of the Methods regarding the Ontario health care system.

*Reviewer #2: This study is interesting for several reasons:
- large samples of people
- lack of data on care and hospitalizations for people in prison and after release
- comparison with the general population*
We appreciate this feedback.

*However, for the method, why the 3 groups do not have the same number of people, since the objective was to compare them.*
Thank you for this comment. We have revised the analyses so that there are now four exposure groups: (i) people in prison, (ii) people in the general population who were matched to people in prison, (iii) people recently released from prison, and (iv) people in the general population who were matched to people recently released from prison. The number of people in groups (i) and (ii) are equal, and the number of people in groups (iii) and (iv) are equal.

*For the results, it is a little difficult to understand why there is such a difference between readmission and return to acute care. Information on the reasons for using non-hospitalized emergencies would deserve to be developed. Interpretation is possibly very much related to the functioning of the health care system in Ontario.*

As noted, we have decided to remove the content on return to acute care, in order to simplify the paper. We have also commented in the limitations section of the Discussion (third paragraph) on our inability to examine reasons for readmission, which may include issues in access to care in emergency departments, and in the final paragraph of the Discussion we noted the need for future research on this topic.

*Minor comments:
Introduction
- Line 62: abbreviation “emergency department (ED)” already indicated line 46*
We have revised this content, and also reviewed the manuscript to ensure consistent use of abbreviations.

*Methods
- Line 84: define ICES*

As per [www.ices.on.ca/About-ICES/New-name-and-logo](http://www.ices.on.ca/About-ICES/New-name-and-logo), ICES is not an acronym but rather the full name of the institute that houses health administrative data for Ontario.
