# Supplementary material for: Thirty-day readmission after medical-surgical hospitalization for people who experience imprisonment in Ontario, Canada: A retrospective cohort study
Source: PLoS One. 2020 Jan 10;15(1):e0227588. doi: 10.1371/journal.pone.0227588 (PMC6953830; doi:10.1371/journal.pone.0227588)
Supplement: Attachment — Submitted filename: Response to reviewers November 27 2019.docx [file pone.0227588.s004.docx]

Response to reviewers
 *Reviewer #1: The authors addressed most of my comments and the current version is improved. I still have some comments, listed below (page numbers refer to the manuscript with track change).*

*1. p. 8 lines 131-138: Please explain how you matched groups (e.g., software).*

We used SAS Enterprise Guide version 7.1 for matching and for all analyses. We have added this information to the end of the Analyses section of the Methods.

*2. Table 1: There is a mistake with the sample size of the matched group for released prisoners (n=1,286 and not n=1,548).*
We apologize for this error, and we have corrected the number.

*3. p. 8 line 134: Please define what is case-mix group of the index hospitalization.*
We have added this information in the last paragraph of the Exposure Group section of the Methods.

*4. Information is still needed on the variables: ADG and Ontario Mental Health and Addictions Scorecard and Evaluation Framework: are those variable summary scores? Continuous? What range? Etc. Please explain more clearly that primary care and ED use are variables (and how they were assessed).*
We have explained that we calculated the total score for ADGs and that the diagnoses as per the Ontario Mental Health and Addictions Scorecard were binary variables (yes/no). We have described primary care use and emergency department use in the second paragraph of the Covariates section of the Methods, and explained that these were binary variables (yes/no).

*5. Please avoid using too many abbreviations, the manuscript is quite hard to follow (se for example sentence p.9 line 156-160: 5 abbreviations).*
In several places, we have replaced abbreviations with full names, including ED, AMA, CIHI, DAD, NACRS, and OHIP.

*6. Discussion: Please also discuss the effect size of the decrease in hospital readmission for prisoners recently released from prison. Maybe it is not clinically relevant.*
We have provided content in the first paragraph of the Discussion regarding the absolute and relative difference in hospital readmission for people in prison and for people recently released from prison. We have also added content to the beginning of the fifth paragraph of the Methods to discuss the absolute difference in risk of hospital readmission.

*7. Reviewer #2: You have decided to cut content regarding the 7-day period, since your focus
is on 30-day readmission rates and your regression analyses are focused on the 30-day period, and in
consideration of sample size issues. You must cut the 7-day data in Table 1 in post discharge section (primary care and emergency department care) and line 401.*

We think it is important to leave in data on primary care and emergency department care in the week after release, as these data may be information to readers who are interested to understand early access to health care post-discharge. People are often advised to follow up with primary care post-discharge, so accessing primary care in that period could indicate accessing appropriate care. ED care in the week after discharge could indicate unmet care needs in the week post-discharge. We have added this information in the last paragraph of the Covariates section in the Methods.
